# Associations of serum cotinine and dietary inflammatory and antioxidant profiles with appendicular skeletal muscle mass in US adults: A cross-sectional study of data from NHANES 2011–2018

**DOI:** 10.18332/tid/222623

**Published:** 2026-07-16

**Authors:** Shifu Bao, Nai Mu, Shuxing Xing, Zheng Zhou

**Affiliations:** 1 Department of Orthopedics, The Fifth People's Hospital Affiliated to Chengdu University of Traditional Chinese Medicine, Chengdu, China

**Keywords:** appendicular skeletal muscle mass, tobacco exposure, serum cotinine, Dietary Inflammatory Index, Composite Dietary Antioxidant Index

## Abstract

**INTRODUCTION:**

Tobacco exposure accelerates biological aging, but its specific association with appendicular skeletal muscle mass – and the potential modifying role of dietary patterns – remains incompletely characterized. This study aimed to investigate the association between serum cotinine and appendicular skeletal muscle mass index (ASMI), and to evaluate the mediating and modifying roles of dietary inflammatory and antioxidant profiles.

**METHODS:**

This secondary analysis of cross-sectional data from the National Health and Nutrition Examination Survey (NHANES) 2011–2018 included 10291 US adults. Serum cotinine and dual-energy X-ray absorptiometry-derived ASMI were measured. Dietary quality was evaluated using the Dietary Inflammatory Index (DII) and Composite Dietary Antioxidant Index (CDAI). Associations were evaluated using multivariable survey-weighted generalized linear models, restricted cubic splines (RCS), and mediation analyses.

**RESULTS:**

In fully adjusted models, higher log10-transformed serum cotinine was independently associated with decreased ASMI (β= -0.02; 95% CI: -0.03 – -0.01; p=0.02). RCS analysis revealed a significant non-linear association (p for non-linearity <0.05). Mediation analyses demonstrated that DII and CDAI accounted for 30.6% and 13.3% of the total association, respectively. Furthermore, this inverse association was pronounced among participants with pro-inflammatory diets (β= -0.08, 95% CI: -0.156 – -0.004, p<0.001) but was attenuated and no longer statistically significant among those consuming anti-inflammatory and antioxidant-rich diets.

**CONCLUSIONS:**

Higher serum cotinine is independently associated with decreased ASMI. This association is partially mediated by dietary inflammatory and antioxidant profiles, and is significantly modified by overall dietary quality. Optimizing nutritional profiles may offer a potential strategy to mitigate tobacco-associated skeletal muscle depletion, though prospective validation is warranted.

## INTRODUCTION

Tobacco use and secondhand smoke (SHS) exposure are leading preventable causes of global morbidity and mortality^1^. In addition to established cardiopulmonary diseases – such as chronic obstructive pulmonary disease, cardiovascular events, and pulmonary malignancies – evidence indicates that tobacco exposure is associated with systemic toxicity^2^. This includes adverse effects on skeletal muscle, bone homeostasis, and neuroendocrine function^3,4^.

Accurate measurement of tobacco exposure is critical in epidemiological studies. Self-reported smoking data are subject to recall and reporting biases, and they often underestimate SHS exposure in non-smokers^5^. Consequently, researchers increasingly use objective biochemical markers. Serum cotinine is currently established as a standard biomarker for assessing systemic tobacco exposure^6^.

Cotinine, the primary hepatic metabolite of nicotine, has a half-life of 16–20 hours, compared to 2–4 hours for nicotine^7^. This stability minimizes the impact of episodic smoking on measured levels. Quantified via isotope-dilution high-performance liquid chromatography coupled with atmospheric pressure chemical ionization tandem mass spectrometry (ID HPLC-APCI MS/MS), serum cotinine provides a reliable measure of recent tobacco exposure^8^.

Serum cotinine concentrations are more stable than urinary levels, making serum the preferred matrix for population-based assessments. Elevated serum cotinine is associated with adverse outcomes in multiple systems, including neurocognitive function^9^, endocrine regulation^10^, and bone metabolism^6^.

Tobacco smoke generates reactive oxygen species (ROS) and reduces endogenous antioxidant defenses^11,12^. This redox imbalance can damage cellular macromolecules and activate pro-inflammatory signaling pathways, contributing to systemic tissue damage^13^. Experimental studies indicate that antioxidant interventions may mitigate nicotine-induced oxidative damage^14^, suggesting that oxidative stress is a key mechanism in tobacco-related pathology^15^.

Skeletal muscle is a critical determinant of metabolic health, physical function, and overall survival^11^. The age-related decline in muscle mass and function – sarcopenia – is associated with an increased risk of falls, fractures, disability, and mortality^16^.

Skeletal muscle relies on mitochondrial networks to meet its energy demands^17^. Tobacco smoke impairs mitochondrial respiration, reduces ATP synthesis, and increases ROS production, disrupting cellular energy homeostasis^18^. Additionally, it suppresses muscle protein synthesis and activates myostatin- and ubiquitin-proteasome-mediated proteolytic pathways, leading to protein catabolism^19^.

Chronic low-grade systemic inflammation activates NF-κB and MAPK signaling pathways in skeletal muscle^20^. Tobacco smoke exacerbates this inflammation via mechanisms such as HMGB1-mediated pyroptosis, contributing to myofiber degradation and muscle atrophy^21^. These pathways provide a biological basis for the association between tobacco exposure and skeletal muscle loss.

Diets with high inflammatory potential, measured by the Dietary Inflammatory Index (DII), are associated with lower muscle strength, reduced muscle mass, and increased sarcopenia risk^22^. Conversely, antioxidant-rich diets, assessed by the Composite Dietary Antioxidant Index (CDAI), may protect against oxidative damage in myofibers^23^. However, the interaction between diet and tobacco exposure on skeletal muscle remains unclear.

We hypothesized that dietary patterns modify the association between tobacco exposure and skeletal muscle mass. Specifically, we expected a stronger inverse association between serum cotinine and appendicular skeletal muscle mass index (ASMI) among individuals with pro-inflammatory diets, and an attenuated association among those with antioxidant-rich diets.

This study aimed to examine the association between serum cotinine and ASMI in a nationally representative sample of US adults. Additionally, we evaluated whether dietary inflammatory and antioxidant profiles mediate or modify this association.

## METHODS

**Figure 1:**
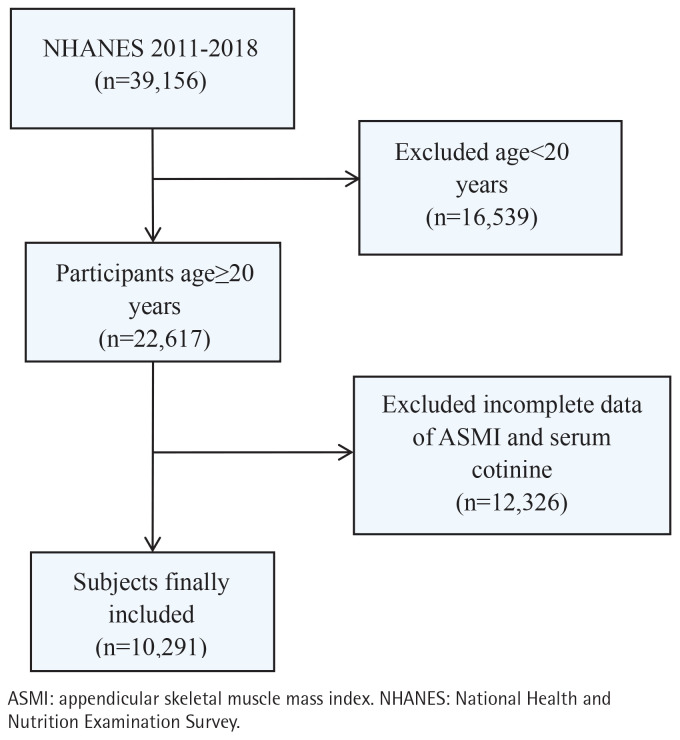
Flowchart for inclusion of participants, United States, NHANES 2011–2018 (N=10291)

This study is a pooled secondary data analysis of cross-sectional data from the National Health and Nutrition Examination Survey (NHANES), conducted in the United States across four cycles (2011–2018). NHANES uses a complex, multistage probability sampling design to provide a nationally representative sample of the non-institutionalized US civilian population. The National Center for Health Statistics Research Ethics Review Board approved all protocols, and all participants provided written informed consent. Among the 39156 individuals initially enrolled, we excluded participants younger than 20 years (n=16539), individuals without appendicular skeletal muscle mass measurements (n=11869), and those missing data on serum cotinine (n=457). Consequently, the final analytic sample consisted of 10291 participants aged ≥20 years. The process of participant selection is illustrated in Figure 1.

### Study variables

*Muscle mass assessment*

Appendicular skeletal muscle mass (ASM) was measured using dual-energy X-ray absorptiometry (DXA) as the sum of lean mass in the upper and lower extremities. The ASMI was calculated by dividing ASM by height squared (kg/m^2^). Sarcopenia was defined using standard gender-specific ASMI thresholds: <7.0 kg/m^2^ for males and <5.5 kg/m^2^ for females^24,25^.

*Serum cotinine*

Venous blood samples were collected at NHANES Mobile Examination Centers (MECs). Serum cotinine concentrations were measured using ID HPLC-APCI MS/MS.

*Dietary inflammatory index and composite dietary antioxidant index*

Dietary inflammatory potential was assessed using the DII. The DII was calculated based on 27 available dietary components, a validated approach comparable to the original 45-parameter model^26^. A DII score ≥0 indicates a pro-inflammatory diet, while a score <0 indicates an anti-inflammatory diet^27^. Dietary antioxidant capacity was evaluated using the CDAI^28^. The CDAI includes six antioxidants: selenium, zinc, carotenoids, and vitamins A, C, and E. The intake of each nutrient was standardized and summed to calculate the total CDAI score.

To examine the combined association of dietary inflammation and antioxidant intake, participants were categorized into three groups: 1) Pro-inflammatory and Pro-oxidative Diet (PIPOD): DII ≥0 and CDAI <0; 2) Mixed Inflammatory/Oxidative Diet (MIOD): DII <0 and CDAI <0, or DII ≥0 and CDAI >0; and 3) Anti-inflammatory and Anti-oxidative Diet (AIAOD): DII <0 and CDAI >0.

### Covariates

We adjusted for potential confounders to evaluate the independent association between serum cotinine and skeletal muscle mass. Covariates included sociodemographic variables: age, gender(male, female), race (American, White, Black, other), marital status (married/living with a partner, widowed/ divorced/separated/never married), education level (lower than 12th grade/high school graduate or equivalent/some college or AA degree/college graduate or higher), poverty income ratio (PIR) and behavioral factors: smoking status (never, former and current), drinking status (never, former, mild, moderate, heavy), physical activity (yes, no). Physical activity (PA) was measured in metabolic equivalent (MET) minutes per week and categorized as meeting (≥600 MET-minutes/week) or not meeting adult activity guidelines^16^. Clinical variables included body mass index (BMI, kg/m^2^), hypertension (yes, no), diabetes mellitus (yes, no), and cardiovascular disease (CVD; yes, no). Biochemical covariates included total protein (g/dL), blood urea nitrogen (mg/dL), serum creatinine (mg/dL), serum creatinine (mg/dL), alkaline phosphatase (U/L), and serum phosphorus (mg/dL). Demographic and behavioral variables were self-reported via a standardized questionnaire, while biochemical markers were measured through laboratory tests.

### Statistical analysis

Statistical analyses incorporated appropriate survey weights, strata, and primary sampling units according to Centers for Disease Control and Prevention (CDC) guidelines. Multi-year Mobile Examination Center (MEC) survey weights were calculated by dividing the 2-year weights by the number of combined cycles.

Baseline characteristics are presented as weighted means with standard errors (SE) for continuous variables, and as weighted frequencies and percentages (%) for categorical variables. Differences between groups were evaluated using design-adjusted Student’s t-tests or Rao-Scott chi-squared tests.

Survey-weighted generalized linear models were used to evaluate the association between serum cotinine and ASMI. Three models were constructed: Model 1 was unadjusted. Model 2 was adjusted for age, gender, and race. Model 3 was adjusted as for Model 2 plus marital status, education level, poverty-to-income ratio (PIR), smoking status, alcohol consumption, physical activity, hypertension, diabetes, cardiovascular disease, BMI, and all biochemical covariates. Serum cotinine was analyzed as both a continuous (log10-transformed) and categorical (tertiles) variable.

Restricted cubic spline (RCS) regressions with three knots (10th, 50th, and 90th percentiles) were used to explore non-linear dose-response associations between log10-transformed serum cotinine and ASMI.

Subgroup analyses were conducted across dietary categories (PIPOD, MIOD, AIAOD). Multiplicative interactions were tested by introducing cross-product terms between serum cotinine and dietary categories into the fully adjusted models.

Mediation analyses with 5000 bootstrap resamples (*mediation* package in R) were performed to estimate the mediating roles of DII and CDAI in the association between serum cotinine and ASMI. All statistical analyses were conducted using R version 4.1.3 (R Foundation for Statistical Computing, Vienna, Austria). Statistical significance was defined as a two-sided p<0.05.

## RESULTS

### Baseline characteristics of participants

Table 1 summarizes the baseline characteristics of the 10291 participants (weighted mean age, 39.39 ± 0.25 years; 50.18% male). Participants in the highest serum cotinine tertile were younger and more likely to be male compared to those in the lowest tertile. Higher cotinine levels were also associated with lower socioeconomic status, higher level of education, and a higher prevalence of frequent alcohol consumption. Nutritionally, individuals in the highest cotinine tertile exhibited more pro-inflammatory and fewer antioxidant-rich dietary patterns. Biochemically, this group demonstrated profiles indicative of greater oxidative stress and systemic inflammation (all p<0.05)

**Table 1:** Characteristics of the study participants according to serum cotinine tertile levels (ng/mL), United States, NHANES 2011–2018 (N=10291)

*Characteristics*	*Total* *n (%)*	*T1* *n (%)*	*T2* *n (%)*	*T3* *n (%)*	p*
**Age** (years), mean ± SE	39.39 ± 0.25	41.10 ± 0.36	38.42 ± 0.38	38.33 ± 0.32	<0.0001
**Gender**					<0.0001
Female	5228 (49.82)	2027 (55.85)	1824 (52.49)	1377 (40.36)	
Male	5063 (50.18)	1414 (44.15)	1597 (47.51)	2052 (59.64)	
**Race**					<0.0001
Black	2120 (10.99)	396 (5.63)	765 (12.97)	959 (15.32)	
Mexican American	1559 (10.65)	750 (13.19)	500 (11.51)	309 (6.90)	
Other	3030 (17.03)	1149 (17.34)	1180 (20.56)	701 (13.35)	
White	3582 (61.33)	1146 (63.84)	976 (54.97)	1460 (64.42)	
**Education level**					<0.0001
Lower than 12th grade	1877 (12.96)	521 (8.77)	550 (11.38)	806 (19.28)	
High school graduate or equivalent	2231 (21.53)	507 (13.99)	687 (20.60)	1037 (31.11)	
Some college or AA degree	3373 (33.21)	1007 (28.94)	1164 (36.05)	1202 (35.46)	
College graduate or higher	2810 (32.30)	1406 (48.29)	1020 (31.97)	384 (14.15)	
**Marital status**					<0.0001
Divorced	946 (9.29)	261 (7.18)	285 (8.57)	400 (12.41)	
Living with partner	1143 (10.58)	246 (6.29)	363 (11.15)	534 (15.02)	
Married	5028 (51.68)	2146 (65.94)	1683 (49.39)	1199 (37.37)	
Never married	2670 (24.54)	661 (17.99)	930 (27.22)	1079 (29.59)	
Separated	363 (2.74)	84 (1.72)	120 (2.64)	159 (4.02)	
Widowed	141 (1.15)	43 (0.89)	40 (1.02)	58 (1.59)	
**Smoking status**					<0.0001
Never	6267 (58.82)	2859 (80.44)	2689 (75.33)	719 (18.33)	
Former	1728 (19.60)	563 (18.92)	688 (23.21)	477 (16.98)	
Current	2296 (21.56)	19 (0.59)	44 (1.46)	2233 (64.68)	
**Drinking status**					<0.0001
Never	1065 (7.71)	555 (8.97)	281 (12.77)	341 (10.84)	
Former	2479 (27.28)	500 (19.29)	663 (21.92)	1329 (43.65)	
Mild	3387 (36.95)	1204 (39.45)	1359 (32.45)	819 (24.27)	
Moderate	1789 (18.82)	606 (19.13)	582 (20.22)	789 (17.34)	
Heavy	1571 (9.23)	576 (13.17)	536 (12.65)	151 (3.91)	
**Diabetes**					0.05
No	1171 (8.91)	413 (8.43)	404 (9.52)	354 (8.90)	
Yes	9120 (91.09)	3028 (91.58)	3017 (90.47)	3075 (91.10)	
**Hypertension**					<0.001
No	7392 (72.93)	2533 (74.47)	2494 (74.64)	2365 (69.53)	
Yes	2899 (27.07)	908 (25.53)	927 (25.36)	1064 (30.47)	
**Cardiovascular diseases**					<0.0001
No	9897 (96.75)	3353 (98.18)	3318 (97.30)	3226 (94.59)	
Yes	394 (3.25)	88 (1.82)	103 (2.70)	203 (5.41)	
**Physical activity**					0.01
No	2963 (28.32)	1131 (22.22)	1060 (20.76)	942 (14.41)	
Yes	7328 (71.68)	2310 (77.78)	2361 (79.24)	2487 (85.59)	
	***Mean ± SE***	***Mean ± SE***	***Mean ± SE***	***Mean ± SE***	
**PIR**	2.94 ± 0.05	3.45 ± 0.06	2.93 ± 0.05	2.38 ± 0.06	<0.0001
**Clinical measurements**					
BMI (kg/m²)	28.68 ± 0.13	28.56 ± 0.17	29.17 ± 0.20	28.36 ± 0.15	<0.001
Alkaline phosphatase (U/L)	67.08 ± 0.41	65.34 ± 0.55	66.74 ± 0.57	69.42 ± 0.61	<0.0001
Serum creatinine (mg/dL)	0.85 ± 0.00	0.84 ± 0.01	0.85 ± 0.01	0.87 ± 0.01	<0.001
Total protein (g/dL)	7.14 ± 0.01	7.14 ± 0.01	7.17 ± 0.01	7.11 ± 0.02	<0.001
Serum uric acid (mg/dL)	5.31 ± 0.02	5.18 ± 0.03	5.36 ± 0.03	5.41 ± 0.04	<0.0001
Blood urea nitrogen (mg/dL)	12.79 ± 0.08	13.37 ± 0.13	12.92 ± 0.10	12.02 ± 0.11	<0.0001
Serum phosphorus (mg/dL)	3.72 ± 0.01	3.71 ± 0.01	3.72 ± 0.01	3.74 ± 0.01	0.41
DII	1.36 ± 0.04	1.12 ± 0.06	1.24 ± 0.06	1.74 ± 0.05	<0.0001
ASMI	7.95 ± 0.03	7.80 ± 0.04	8.01 ± 0.04	8.06 ± 0.05	<0.0001
CDAI	0.84 ± 0.07	1.22 ± 0.11	1.01 ± 0.12	0.24 ± 0.09	<0.0001

SE: standard error. BMI: body mass index. PIR: poverty income ratio. DII: Dietary Inflammatory Index. CDAI: Composite Dietary Antioxidant Index. ASMI: appendicular skeletal muscle mass index . T1: cotinine level <0.018 ng/mL. T2: 0.018≤ cotinine level ≤0.492 ng/mL. T3: cotinine level >0.492 ng/mL.

* Calculated using weighted chi-squared tests for categorical variables and one-way analysis of variance (ANOVA) for continuous variables.

### Association between serum cotinine and ASMI

Table 2 presents the associations between serum cotinine and ASMI. In the fully adjusted model (Model 3), higher log10-transformed serum cotinine was significantly associated with decreased ASMI (β= -0.02; 95% CI: -0.03 – -0.01; p=0.02). Categorical analyses using cotinine tertiles yielded consistent results; compared to the lowest tertile (T1), participants in the highest tertile (T3) had significantly lower ASMI (β= -0.07; 95% CI: -0.11 – -0.02; p<0.01).

**Table 2:** Association between the serum cotinine and appendicular skeletal muscle mass index (ASMI), multivariable linear regression, United States, NHANES 2011–2018 (N=10291)

*Serum cotinine (ng/mL)*	*β (95% CI) p*		
	*Model 1*	*Model 2*	*Model 3*
**log10-transformed cotinine**	0.04 (0.01–0.04) <0.01	-0.05 (-0.07 – -0.03) <0.0001	-0.02 (-0.03–0.01) 0.02
**Tertiles**			
T1 (ref.)			
T2	0.21 (0.11–0.32) <0.001	0.08 (-0.02–0.17) 0.12	-0.03 (0.08–0.02) 0.21
T3	0.26 (0.14–0.39) <0.0001	-0.13 (-0.23 – -0.03) <0.01	-0.07 (-0.11 – -0.02) <0.01
p for trend	<0.0001	<0.0001	0.32

Model 1 was unadjusted. Model 2 adjusted for gender, age, and race; Model 3 adjusted as for Model 2 plus marital status, education level, poverty income ratio, smoking status, drinking status, physical activity, hypertension, diabetes, cardiovascular diseases, body mass index, and biochemical markers (total protein, blood urea nitrogen, serum creatinine, serum calcium, alkaline phosphatase, and serum phosphorus). T1: cotinine level <0.018 ng/mL. T2: 0.018≤ cotinine level ≤0.492 ng/mL. T3: cotinine level >0.492 ng/mL. Analyses incorporated NHANES survey sampling weights to account for the complex survey design.

### Mediation analyses

**Figure 2:**
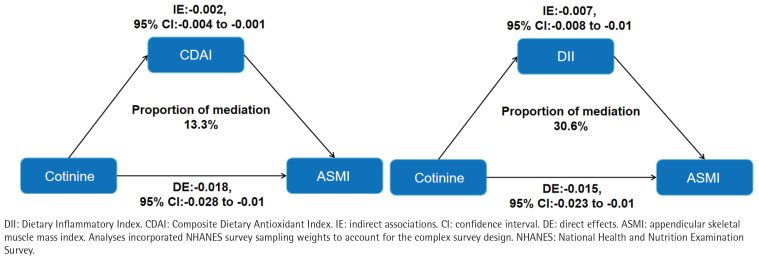
Indirect associations of DII and CDAI in the association between serum cotinine and ASMI, United States, NHANES 2011–2018 (N=10291)

**Figure 3:**
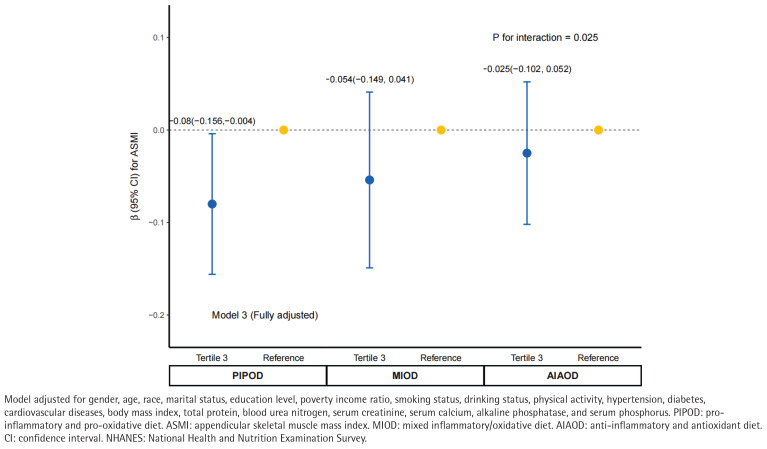
Anti-inflammatory or antioxidant diets counteract serum cotinine-associated ASMI, United States, NHANES 2011–2018 (N=10291)

Parallel mediation analyses indicated that both CDAI and DII significantly mediated the association between serum cotinine and ASMI (Figure 2). The indirect effect via CDAI was -0.003 (95% CI: -0.004–0.000; p<0.001), accounting for 13.3% (95% CI: 7.8–23.0) of the total association. The indirect effect via DII was -0.007 (95% CI: -0.008 – -0.001; p<0.001), mediating a larger proportion (30.6%; 95% CI: 20.5–52.0) of the total association compared to CDAI. Furthermore, we examined the modifying influence of composite dietary profiles on this relationship. The inverse association remained significant among participants with a pro-inflammatory and pro-oxidative diet (β= -0.08; 95% CI: -0.156 – -0.004, p<0.001), whereas it was attenuated and no longer statistically significant among those with an anti-inflammatory and anti-oxidative diet (β= -0.025; 95% CI: -0.102–0.052, p>0.05). The interaction between serum cotinine and dietary profiles was statistically significant (p for interaction=0.025) (Figure 3).

### Subgroup analyses

**Figure 4:**
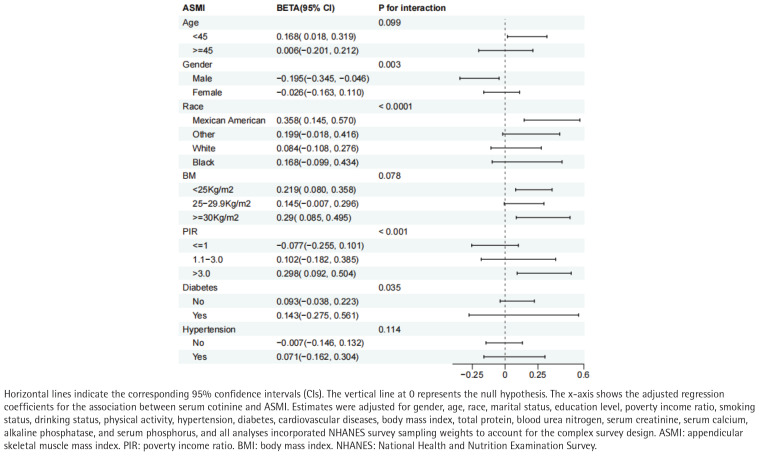
Subgroup analysis for the association between serum cotinine and ASMI, United States, NHANES 2011–2018 (N=10291)

Stratified analyses identified significant interactions between serum cotinine and gender (p for interaction=0.003), race (p<0.001), PIR (p<0.001), and diabetes status (p=0.035). Specifically, the inverse association between serum cotinine and ASMI was more pronounced among males, Mexican Americans, individuals at both socioeconomic extremes, and those with diabetes (Figure 4).

### Restricted cubic splines

Restricted cubic spline analysis revealed a significant non-linear association between log10-transformed serum cotinine and ASMI (p for non-linearity <0.05) (Supplementary file Figure 1). ASMI decreased with increasing cotinine concentrations up to an inflection point of approximately -1.0 (equivalent to 0.1 ng/ mL), beyond which it plateaued.

## DISCUSSION

In this nationally representative cross-sectional study of US adults, higher serum cotinine was independently associated with decreased ASMI. Mediation analyses indicated that the DII and CDAI accounted for 30.6% and 13.3% of this association, respectively. Furthermore, this inverse association was pronounced among individuals with pro-inflammatory and pro-oxidative diets, but was no longer significant among those consuming anti-inflammatory and anti-oxidative diets.

Our findings indicate that dietary inflammatory and antioxidant profiles partially mediate the association between serum cotinine and ASMI. While previous observational studies link pro-inflammatory diets to reduced muscle mass^29^ and antioxidant-rich diets to favorable muscle outcomes^23^, we position these dietary indices as intermediate pathways in tobacco-associated muscle depletion.

Several biological mechanisms may explain the observed associations. First, cigarette smoke generates severe oxidative stress by depleting endogenous antioxidant reserves^30^ and inducing mitochondrial dysfunction, which reduces ATP availability and amplifies ROS production^31^. Second, this redox imbalance activates stress-sensitive signaling cascades (e.g. p38 MAPK and NF-κB) that upregulate the ubiquitin-proteasome system – specifically E3 ubiquitin ligases (MuRF1 and MAFbx) – driving uncompensated myofibrillar protein breakdown^32,33^. Finally, tobacco toxicants impair satellite cell function, blunting skeletal muscle regenerative capacity and recovery from injury or disuse^33^. Collectively, this dual burden of accelerated catabolism and impaired regeneration drives the progressive muscle depletion associated with systemic tobacco exposure.

Our findings suggest that nutritional quality significantly modulates the myocellular response to tobacco toxicants. Pro-inflammatory diets amplify tobacco-triggered systemic inflammation^29^. Conversely, dietary antioxidants scavenge ROS and chelate redox-active metals, limiting oxidative damage to myocellular proteins and lipids^34^. Additionally, micronutrients (e.g. zinc and selenium) serve as essential cofactors for endogenous antioxidant enzymes (e.g. superoxide dismutase and glutathione peroxidase), preserving mitochondrial efficiency^35^.

The attenuation of the cotinine–ASMI association among individuals with high dietary antioxidant intake suggests that optimal nutrition may buffer skeletal muscle against tobacco-related oxidative injury. This aligns with experimental data demonstrating that antioxidant supplementation reduces oxidative stress in tobacco-exposed populations, and with epidemiological evidence linking higher dietary antioxidant scores to reduced sarcopenia risk^11^.

### Strengths and limitations

This study has several strengths. First, using serum cotinine – an objective biomarker – minimizes exposure misclassification compared to self-reported smoking status. Second, the complex, multistage probability design of NHANES, combined with appropriate survey weights, ensures the generalizability of our findings to the US adult population. Third, the simultaneous assessment of the DII and CDAI provides a more comprehensive evaluation of dietary quality than either index alone. Finally, our robust analytical framework – incorporating non-linear modeling, mediation, and stratified analyses – allows for an exploration of dose-response relationships, underlying pathways, and susceptible subpopulations.

Several limitations warrant consideration. First, our cross-sectional design precludes causal inference. Reverse causation remains possible; for instance, individuals with pre-existing low muscle mass might preferentially consume softer, highly processed (pro-inflammatory) foods. Second, despite rigorous covariate adjustment, residual confounding from unmeasured variables – such as specific resistance training protocols, genetic predispositions, or the ‘healthy user’ bias – cannot be completely excluded. Third, excluding participants with missing data may introduce selection bias. Furthermore, serum cotinine reflects only short-term tobacco exposure (preceding several days), potentially misclassifying intermittent smokers. Finally, our primary outcome was restricted to DXA-derived macroscopic muscle mass, lacking complementary assessments of muscle quality (e.g. myosteatosis) or physical function (e.g. grip strength) that could capture early functional decline.

## CONCLUSIONS

In this nationally representative study, higher serum cotinine was independently associated with decreased ASMI. This inverse association was exacerbated by pro-inflammatory diets and attenuated by anti-inflammatory, antioxidant-rich dietary patterns, with DII and CDAI acting as partial mediators. These findings expand the understood scope of tobacco-related harm beyond the classical cardiopulmonary disease spectrum, highlighting skeletal muscle as a vulnerable target. While inherently hypothesis-generating, our results underscore the potential of targeted nutritional interventions to mitigate tobacco-associated muscle deterioration. Prospective cohort studies and randomized controlled trials are warranted to confirm causality and evaluate the clinical efficacy of these dietary strategies.

## Supplementary Material



## Data Availability

The datasets used in this study are available in online repositories. Details including the repository names and accession numbers can be accessed here: https://wwwn.cdc.gov/nchs/nhanes/Default.aspx
