# Supplementary material for: Associations of serum cotinine and dietary inflammatory and antioxidant profiles with appendicular skeletal muscle mass in US adults: A cross-sectional study of data from NHANES 2011–2018
Source: Tob Induc Dis. 2026 Jul 16;24:10.18332/tid/222623. doi: 10.18332/tid/222623 (PMC13377826; doi:10.18332/tid/222623)
Supplement: Supplementary file 1 [file TID-24-111-s1.pdf]

TID
Tobacco Induced Diseases

Supplementary file

© 2026 Bao S. et al.

DOI:
10.18332/tid/222623

The content has been provided by the author(s) and has not been reviewed, verified, or endorsed by European Publishing. It may not have undergone peer review. The views, opinions, and recommendations expressed are solely those of the author(s) and do not necessarily reflect the position of European Publishing. European Publishing accepts no responsibility or liability for any consequences arising from the use of, or reliance on, this content.

Supplementary file Figure S1. Restricted cubic spline analyses of serum cotinine and appendicular skeletal muscle mass index (ASMI), United States, NHANES 2011–2018(n=10,291)

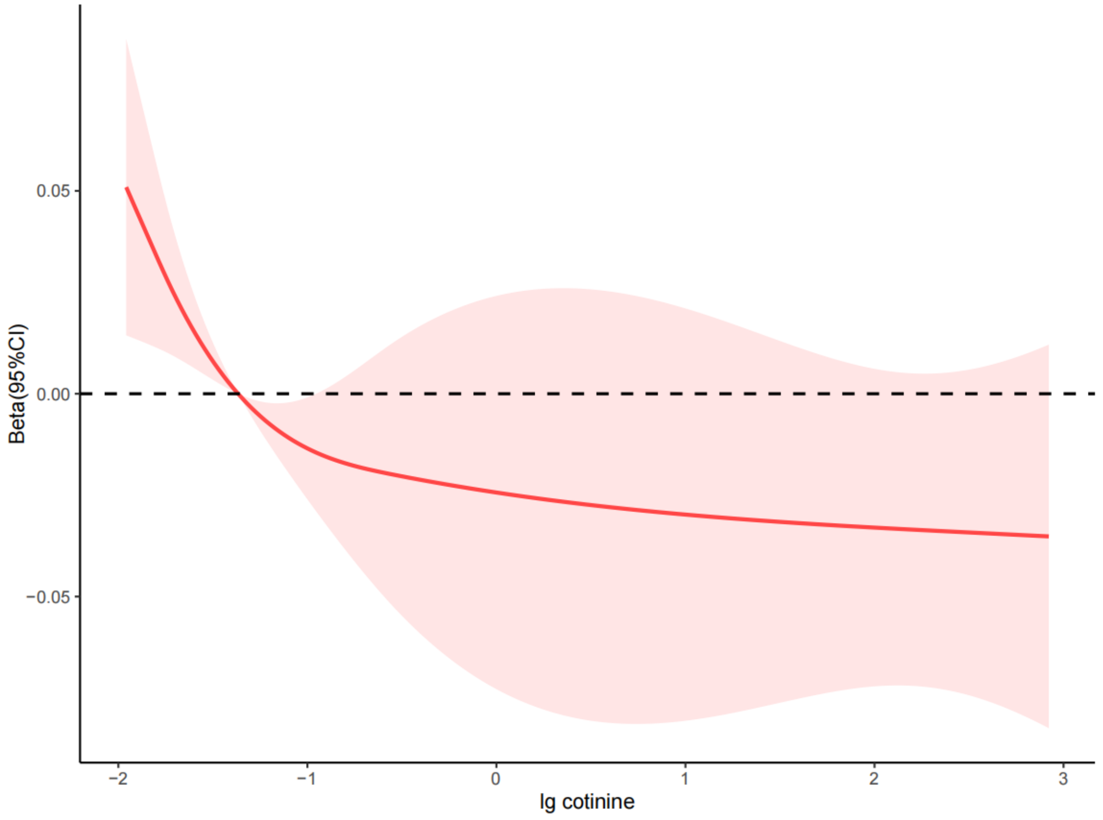

Restricted cubic spline analysis depicting the potential nonlinear associations between serum cotinine and ASMI. Model adjusted for gender, age, and race, marital status, education, poverty income ratio, smoking status, drinking status, physical activity, hypertension, diabetes, cardiovascular diseases, body mass index, and biochemical markers (total protein, blood urea nitrogen, serum creatinine, serum calcium, alkaline phosphatase, and serum phosphorus). Log10-transformed serum cotinine (ng/mL) on the x-axis and ASMI (kg/m²) on the y-axis.P-values for nonlinearity were < 0.05, indicating significant evidence of nonlinear associations.Analyses incorporated NHANES survey sampling weights to account for the complex survey design.NHANES: National Health and Nutrition Examination Survey.
